# Comparative evaluation of microneedling technique and diode laser in gingival depigmentation: A prospective randomized study

**DOI:** 10.4317/jced.64126

**Published:** 2026-06-29

**Authors:** Natasha Singh, Ruchi Pandey, Nipun Dhalla, Lipika Gopal, Diksha Swaroop

**Affiliations:** 1 Department of Periodontology. Manav Rachna Dental College, Sector – 43, Delhi, Suraj Kund Badkhal Rd, Faridabad, Haryana 121010

## Abstract

**Background:**

Gingival hyperpigmentation due to increased melanocyte activity is a common esthetic concern. Although several depigmentation techniques exist, recurrence remains a clinical challenge. Minimally invasive approaches such as diode laser and microneedling with ascorbic acid have gained attention. Aim: To compare gingival re-pigmentation following depigmentation using diode laser and microneedling with ascorbic acid.

**Materials and Methods:**

This randomized controlled study included 32 systemically healthy patients (DOPI 1). Participants were allocated into Group A (diode laser, 940 nm) and Group B (microneedling with topical ascorbic acid). Clinical parameters-Visible Plaque Index (VPI), Modified Gingival Index (MGI), and Dummett-Gupta Oral Pigmentation Index (DOPI)-were recorded at baseline, 3, 6, and 12 months. Pain was assessed using VAS.

**Results:**

Both groups showed significant reductions in DOPI from baseline to 12 months (p = 0.0001). Near-complete depigmentation was observed at 3 months, with mild re-pigmentation at 12 months (0.42 ± 0.34 and 0.47 ± 0.22), without significant intergroup differences (p &gt; 0.05). Microneedling showed higher 24-hour VAS scores, but pain resolved in both groups by day 7. VPI and MGI improved significantly in both groups (p = 0.0001).

**Conclusions:**

Both modalities are effective, safe, and cost-effective, with comparable long-term outcomes. Diode laser demonstrated lower immediate postoperative discomfort.

## Introduction

Gingival aesthetics is a key component of dentofacial harmony, significantly influencing smile perception. Gingival pigmentation, although often physiological, is frequently considered esthetically undesirable and may affect patient confidence. It is multifactorial in origin, involving pigments such as melanin, melanoid, carotene, hemoglobin derivatives, bilirubin, and iron deposits (1). Gingival color is determined by epithelial thickness, vascularity, keratinization, and pigment distribution. Among these, melanin-produced by melanocytes via a tyrosine-dependent pathway and transferred to keratinocytes-is the primary contributor to gingival pigmentation and a major concern in esthetic dentistry (2 , 3). Various techniques have been proposed for gingival depigmentation, including surgical scraping, gingivectomy, electrosurgery, and cryosurgery; however, these are often associated with postoperative discomfort, bleeding, and delayed healing. Diode lasers have gained preference due to their ease of use, selective melanin absorption, and superior hemostasis, although outcomes may vary with technique and parameters (4). Microneedling has recently emerged as a minimally invasive alternative, creating microchannels that enhance topical drug delivery and promote epithelial remodeling. It has shown promising results with favorable healing, minimal discomfort, and low thermal damage (5). The adjunctive use of ascorbic acid (vitamin C) further enhances outcomes by inhibiting tyrosinase, reducing oxidative intermediates, and interrupting melanogenesis, while also supporting collagen synthesis and tissue repair. Despite the growing adoption of diode laser therapy and microneedling with ascorbic acid, direct comparative evidence evaluating their clinical efficacy and long-term outcomes remains limited. In particular, the issue of re-pigmentation continues to be inadequately addressed in existing literature. Therefore, the present study was designed to compare these two modalities in terms of gingival re-pigmentation, postoperative healing, pain perception, and patient-centered outcomes over a 12-month follow-up period.

## Materials and Methods

**Figure 1:**
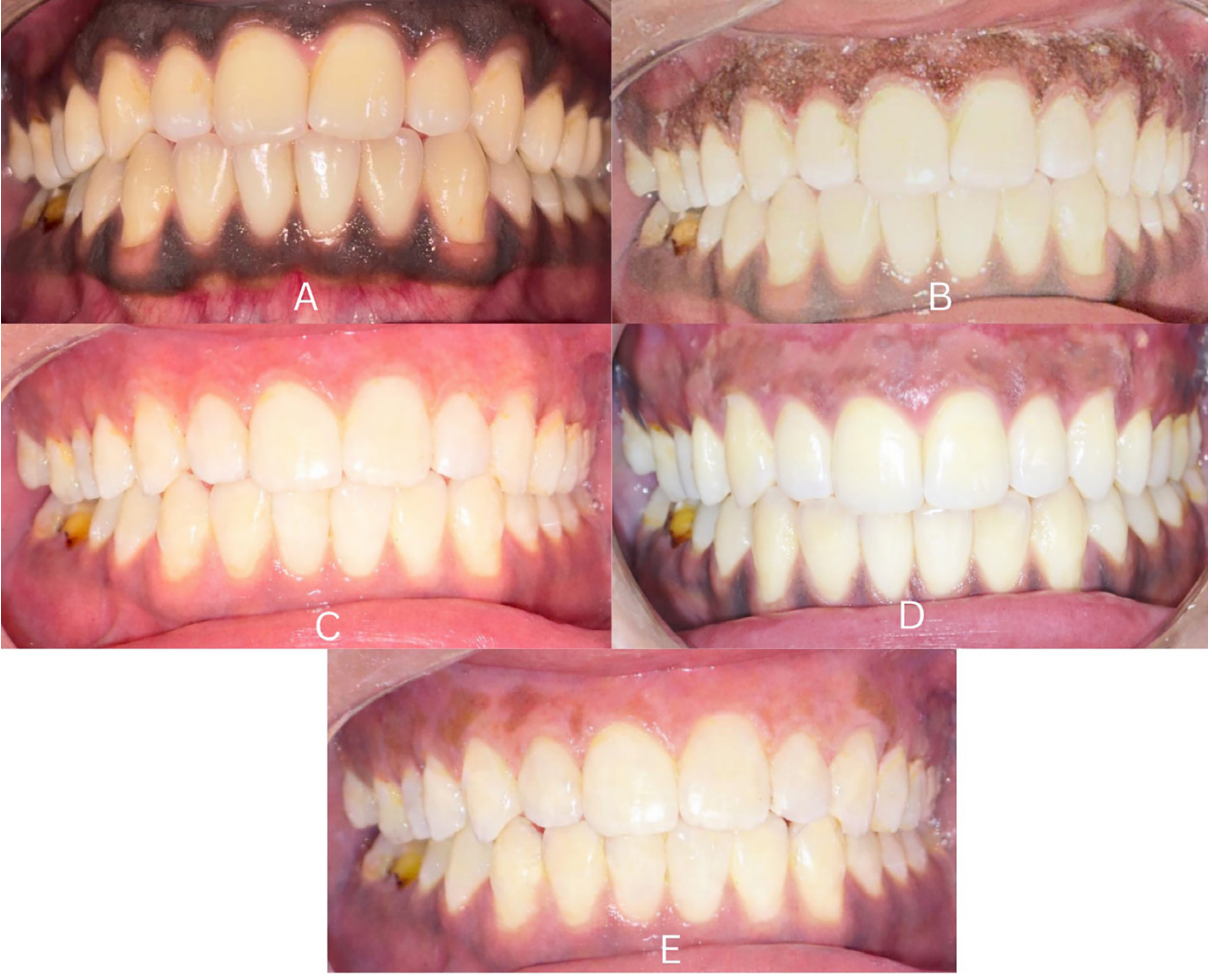
Clinical Parameters Visible Plaque index Modified Gingival Index DOPI at A) Baseline B) Depigmentation using Laser technique. C) 3 months D) 6 months and E) 12 months.

1. Trial Design The present study was designed as a prospective, parallel-arm, randomized controlled clinical study to evaluate and compare the effectiveness of diode laser therapy and microneedling with ascorbic acid in the management of gingival pigmentation. The study was conducted in the Department of Periodontology at Manav Rachna Dental College, SDS, MRIIRS, Faridabad, India. Ethical approval was obtained from the Institutional Ethics Committee (MRDC/IEC/2024/24), and the trial was registered with the Clinical Trial Registry of India (CTRI/2025/06/088632). 2. Participants Participants were recruited from the outpatient Department of Periodontology, Manav Rachna Dental College, Faridabad, India. Patients presenting with gingival hyperpigmentation were screened and diagnosed clinically based on visible melanin pigmentation (DOPI 1) (6). Baseline periodontal status was assessed using the Visible Plaque Index (VPI) (7) and Modified Gingival Index (MGI) (8), and gingival phenotype was measured using transgingival probing with a digital vernier caliper. All participants underwent initial phase therapy, including scaling and oral hygiene instructions, prior to baseline recording. Clinical parameters were recorded at baseline, 3, 6, and 12 months. Postoperative pain was assessed using the Visual Analog Scale (VAS) (9) at 24 hours and 7 days. 3. Eligibility Criteria All participants underwent phase I periodontal therapy, including full-mouth scaling and oral hygiene instructions, and were re-evaluated after two weeks. Only those demonstrating minimal VPI and MGI scores were included. The study adhered to the principles of the Declaration of Helsinki. Participants were systemically healthy individuals aged 18-40 years with physiologic gingival pigmentation (DOPI 1) and adequate gingival phenotype. Subjects with the presence of systemic diseases or medications affecting pigmentation, pregnancy and/or lactation, history of smoking or tobacco use, periodontal therapy within the previous 3 months, and presence of depigmentation disorders such as vitiligo were excluded. 4. Intervention Before surgical intervention, all participants received non-surgical periodontal therapy, including oral hygiene instructions and full-mouth scaling to achieve optimal plaque control. All procedures were performed under local anesthesia using 2% lidocaine with 1:80,000 adrenaline. The surgical site was disinfected extraorally with 5% povidone-iodine, and standard aseptic protocols were followed. Diode laser group: Gingival depigmentation was carried out using a 940 nm diode laser (Biolase Epic®) at a power setting of 0.6 W in continuous contact mode with a pre-initiated 320 m fiber optic tip. The pigmented gingival epithelium was ablated using controlled sweeping, paintbrush-like strokes, starting from the mucogingival junction and extending toward the free gingival margin, including the interdental papillae. The fiber tip was continuously moved in overlapping motions to prevent excessive heat accumulation. The treated surface was intermittently wiped with sterile gauze soaked in normal saline to remove debris and improve visibility. The procedure was continued until complete removal of visible pigmentation was achieved, as shown in Figure 1.

1

**Figure 2:**
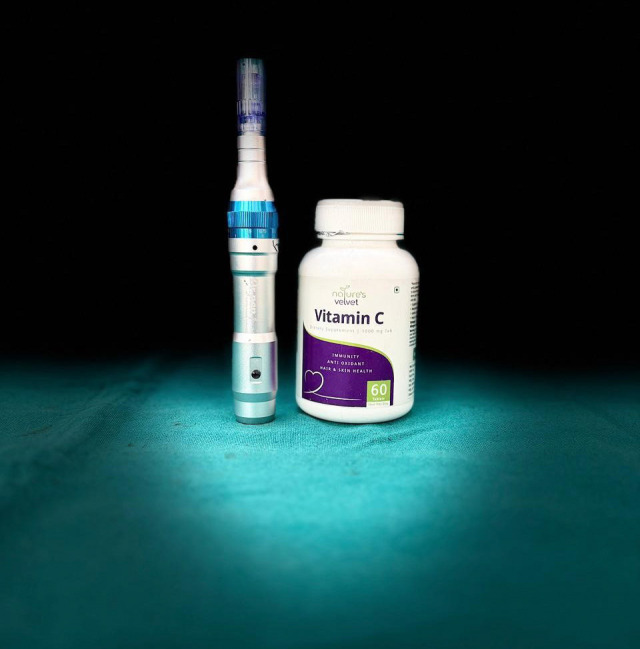
Derma Pen with Vitamin C capsule.

Microneedling group: Depigmentation was performed using a Dermapen device (Dr.Pen Ultima A6®) equipped with 1.5 mm sterile needles (Fig. 2).

2

**Figure 3:**
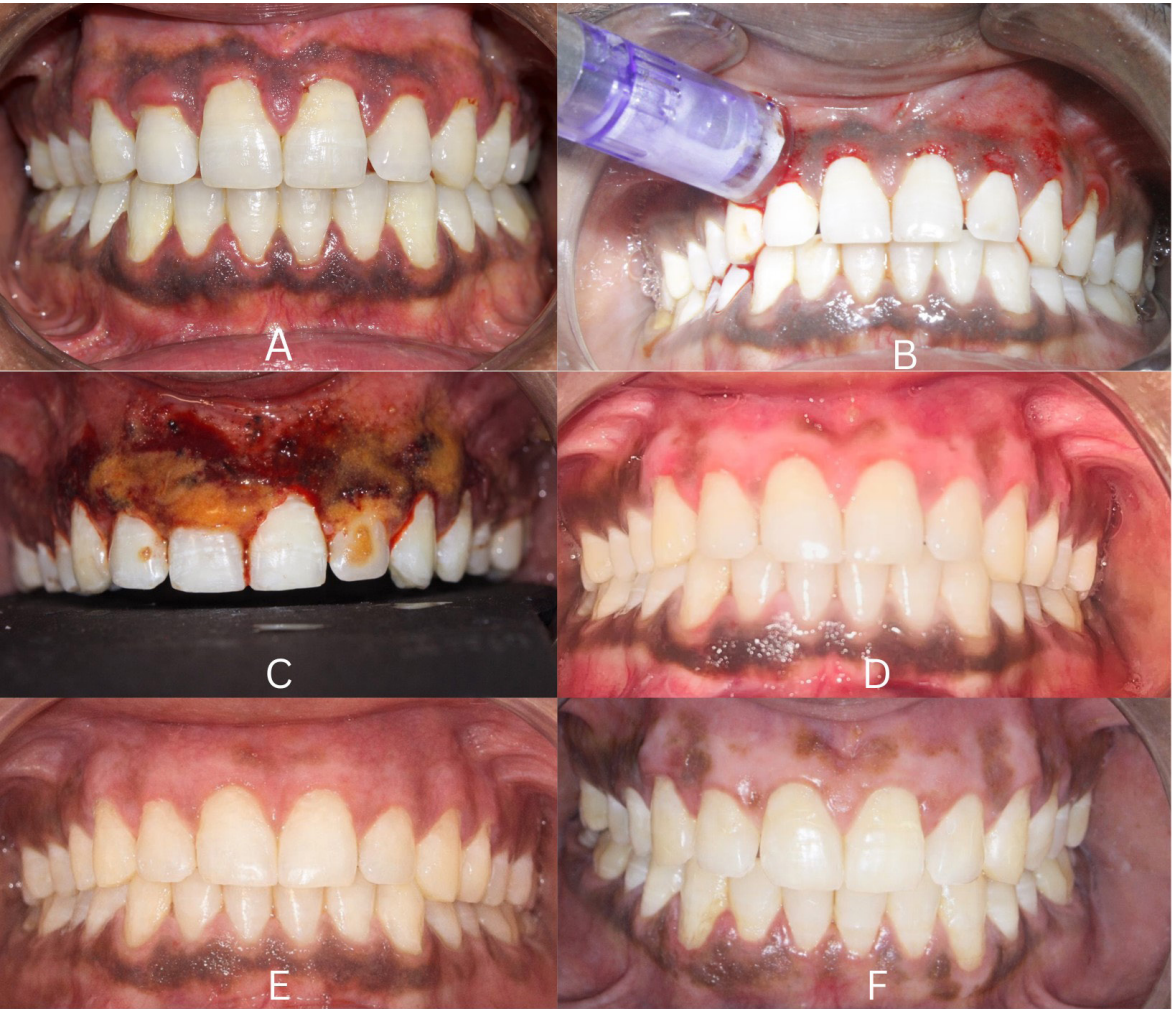
Clinical Parameters Visible Plaque index Modified Gingival Index DOPI at A) Baseline B) Depigmentation using Micro needling technique C) Ascorbic Acid application D) 3 months E) 6 months, F) 12 months.

The device was applied uniformly over the pigmented gingival surface until pinpoint bleeding was observed, indicating adequate penetration and microchannel formation. Following microneedling, topical ascorbic acid (1000 mg/ml) prepared by dissolving vitamin C powder in saline was applied over the treated area and left undisturbed for approximately 10 minutes to enhance trans-epithelial absorption. Then it was gently rinsed with water. No periodontal dressing was placed in both groups. Postoperatively, all participants were instructed to avoid hot, spicy, and acidic foods for 24 hours and to refrain from toothbrushing in the treated area for one day. Standard oral hygiene measures were reinforced thereafter. Patients were advised to report any adverse events such as pain, swelling, or hypersensitivity. Follow-up evaluations were scheduled at 24 hours, 7 days, 3 months, 6 months, and 12 months postoperatively. Clinical parameters were recorded at each visit to assess healing, pain, and pigmentation as shown in Figure 3.

3

**Figure 4:**
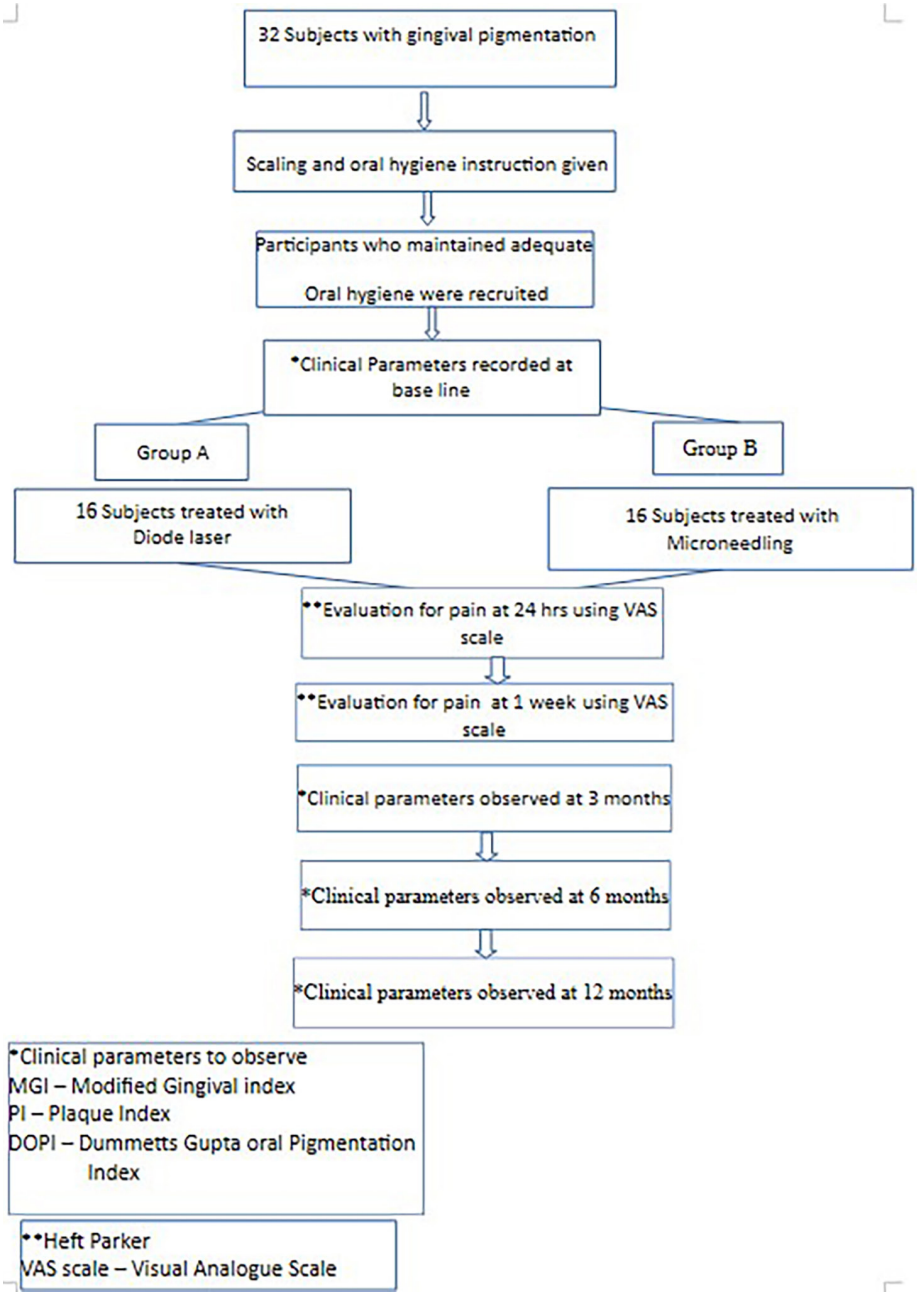
Flowchar to the study.

5. Outcomes The primary outcome was change in gingival pigmentation assessed using the Dummett-Gupta Oral Pigmentation Index (DOPI) (6) at baseline, 3, 6, and 12 months. Secondary outcomes included Visible Plaque Index (VPI), (7) Modified Gingival Index (MGI) (8) ,and Visual Analog Scale (VAS) (9) for pain (24 hours, 7 days). 6. Sample Size The sample size was determined in consultation with a statistician based on the primary outcome (change in DOPI). Due to limited prior comparative data, a pragmatic approach was adopted. A total of 36 participants were recruited to account for an anticipated 10% dropout; 32 participants completed the study and were included in the final analysis. The sample was equally distributed between the two groups following randomization. 7. Randomisation Participants were randomly assigned to the diode laser group (Group A) or the microneedling with ascorbic acid group (Group B) using a computerized randomization sequence. Allocation concealment was ensured using sealed, opaque envelopes, opened at the time of intervention. Due to the nature of the interventions, operator blinding was not feasible. However, the outcome assessor was blinded to group allocation, and standardized clinical protocols were followed to minimize bias. The allocation sequence was securely maintained throughout the study, as shown in Fig. 4.

4

8. Statistical Methods All statistical analyses were performed using SPSS version 25.0 (IBM Corp., Armonk, NY, USA). The distribution of continuous variables was assessed using the Shapiro-Wilk test. Normally distributed data were expressed as mean ± standard deviation (SD). For intragroup comparisons, the paired t-test was used, while intergroup comparisons were performed using the independent (unpaired) t-test. A two-tailed p-value &lt; 0.05 was considered statistically significant, with a 95% confidence interval applied throughout.

## Results

1. Visible Plaque Index (7) Baseline VPI was comparable between Group A (0.96 ± 0.20) and Group B (0.96 ± 0.08) (p = 0.911). Both groups showed significant intragroup reductions (p = 0.0001). At 12 months, VPI decreased to 0.60 ± 0.24 (Group A) and 0.56 ± 0.18 (Group B), with no intergroup differences at any time point (p &gt; 0.05), (Table 1,2).

Table 1

Table 2

2. Modified Gingival Index Baseline MGI was comparable between Group A (1.18 ± 0.32) and Group B (0.99 ± 0.32) (p = 0.098). Both groups showed significant intragroup reductions (p = 0.0001). At 12 months, MGI decreased to 0.61 ± 0.35 (Group A) and 0.46 ± 0.21 (Group B), with no intergroup differences at any time point (p &gt; 0.05), (Table 1,2). 3. Dummett-Gupta Oral Pigmentation Index (6) Baseline DOPI scores were 2.05 ± 0.58 (Group A) and 2.06 ± 0.60 (Group B), confirming equivalence (p = 0.977). Near-complete depigmentation was achieved at 3 months in both groups (Group A: 0.05 ± 0.16; Group B: 0.00 +/- 0.00). Mild re-pigmentation was observed at 6 months (Group A: 0.15 ± 0.28; Group B: 0.07±0.17) and 12 months (Group A:0.42±0.34; Group B:0.47±0.22). Intragroup reductions from baseline remained highly significant at all time points in both groups (p = 0.0001). No statistically significant intergroup differences were observed at 3 months (p = 0.178), 6 months (p = 0.381), or 12 months (p = 0.632), indicating comparable depigmentation efficacy and re-pigmentation patterns (Table 3).

Table 3

4. Visual Analogue Scale (9) At 24 hours, mean VAS scores were 6.44 ± 10.04 (Group A) and 21.69 ±29.03 (Group B). Although microneedling resulted in higher immediate postoperative discomfort, this difference did not reach statistical significance (p = 0.056). By day 7, VAS scores were 0.0 ± 0.0 in both groups, indicating complete pain resolution. Intragroup reductions from 24 hours to 7 days were statistically significant in both groups (Group A: p = 0.022; Group B: p = 0.009). The intergroup difference in the magnitude of VAS change from 24 hours to 7 days was significant (p = 0.001), reflecting the greater initial pain burden in the microneedling group (Table 4).

Table 4

## Discussion

The role of the diode laser in treating oral tissue pathology is well documented, primarily due to its high affinity for pigmented tissues such as hemoglobin and melanin (10). The present randomized controlled study provides 12-month comparative evidence on two minimally invasive approaches to gingival depigmentation. The principal finding is that both diode laser (940 nm) and microneedling with topical ascorbic acid achieve substantial, sustained depigmentation with comparable long-term outcomes, while differing meaningfully in immediate postoperative comfort. The near-complete DOPI reduction at 3 months in both groups is consistent with prior literature. Diode lasers achieve depigmentation through selective photothermal ablation of melanocyte-containing epithelial layers, producing simultaneous coagulation, a bloodless surgical field, and bactericidal effects. Microneedling creates microchannels that enhance trans-epithelial delivery of ascorbic acid, which inhibits tyrosinase, scavenges reactive oxygen species, and promotes reduction of dopaquinone, interrupting melanin synthesis. These mechanistically distinct pathways converge on equivalent clinical depigmentation. The gradual re-pigmentation observed between 6 and 12 months is consistent with melanocyte migration from adjacent tissues. Laser therapy delays repopulation through thermal ablation, whereas microneedling with vitamin C provides transient biochemical inhibition of melanogenesis. This may explain the slightly earlier recurrence in the microneedling group, although the difference was not statistically significant. The absence of significant intergroup differences in DOPI aligns with previous studies. Esmat et al. reported comparable depigmentation outcomes between vitamin C mesotherapy and diode laser, while Singh et al. demonstrated similar long-term efficacy across modalities (11 , 12). The present study extends these findings to a 12-month follow-up. Furthermore, a systematic review by Sanadi et al. supports the role of vitamin C in inhibiting melanogenesis via tyrosinase suppression, reinforcing its clinical utility in managing gingival hyperpigmentation (13). Mostafa Sr et al. found marked reduction in DOPI and Hedin Melanin Index scores after microneedling combined with topical vitamin C, with most patients exhibiting marked pigment clearance from baseline to follow-up (5). Further supporting this, novel evidence from Mansuri et al. demonstrated significant melanin reduction after microneedling with vitamin C across age groups, suggesting that microneedling is a safe and effective depigmenting modality (14). Al-Hajri et al. reported that both conservative injectable vitamin C and scalpel surgical depigmentation produced a significant decline in gingival pigmentation from baseline. Mild recurrence occurred in both groups during extended follow-up; however, overall depigmentation remained statistically significant compared to baseline (15). Higher 24-hour VAS scores in the microneedling group may be attributed to mechanical microtrauma, whereas diode lasers reduce pain through coagulation and sealing of nerve endings. Despite this early difference, both groups showed complete pain resolution by day 7, indicating similar recovery patterns. These findings are consistent with previous studies reporting lower early postoperative pain with the diode laser (11 , 16). Nammour et al. conducted a randomized comparative clinical study evaluating different laser wavelengths for gingival depigmentation. Postoperative pain was assessed using a Visual Analog Scale (VAS) at 24 hours, 3 days, 1 week, and during subsequent follow-ups up to 12 months. The authors reported that although pain scores were numerically different among wavelengths at 24 hours, the differences were not consistently statistically significant (p &gt; 0.05) across all laser groups. By 1 week, pain levels had markedly decreased in all groups, and intergroup differences were generally not statistically significant, indicating comparable short-term discomfort irrespective of wavelength (17). Gopalakrishnan et al. also reported a significant decline in pain from Day 1 to Day 7 in both laser and scalpel groups, with a non-significant intergroup difference at 1 week (18). Both modalities showed significant improvements in VPI and MGI, likely reflecting improved oral hygiene practices rather than a direct treatment effect. No adverse events were observed. To our knowledge, this is the first study comparing long-term re-pigmentation between diode laser and microneedling with ascorbic acid. Comparing the two treatment modalities, micronee-dling with vitamin C promotes collagen induction, potentially increasing gingival thickness, thereby reducing the chances of gingival recession (19) compared to laser ablation. Vitamin C acts as a biocompatible inhibitor of tyrosinase, leading to effective and stable long-term results. Diode laser offers superior immediate comfort and hemostasis, whereas microneedling repre-sents a cost-effective alternative with comparable esthetic outcomes.

## Limitations

The 12-month follow-up limits assessment of long-term re-pigmentation, and the relatively small sample size may affect generalizability. Both procedures are operator-dependent, with potential procedural variability. Microneedling in the interdental papilla region poses technical challenges. Furthermore, the use of index-based assessment methods may reduce precision due to their subjective nature; more objective techniques, such as spectrophotometry or RGB analysis, would provide a more accurate evaluation of re-pigmentation. Further studies with a larger sample size and longer follow-up are warranted to substantiate the current study results.

## Conclusions

Within the study limitations, both diode laser (940 nm) and microneedling with ascorbic acid were effective and safe, with comparable depigmentation and mild re-pigmentation at 12 months. Diode laser demonstrated lower immediate postoperative discomfort. However, mi-croneedling may serve as a cost-effective and more patient-acceptable alternative compared to diode laser therapy. Future research should focus on a large sample size and long-term follow-up to better evaluate the rate and extent of re-pigmentation and to validate the present findings. Clinical decision-making may be guided by factors such as availability, cost, and patient preference.

## Figures and Tables

**Table 1:** Comparison of study groups with respect to Plaque index and Gingival index at different time intervals.

Parameter	Groups	Baseline		3 months		6 months		12 months	
		Mean	SD.	Mean	SD.	Mean	SD.	Mean	SD.
Plaque Index	Group A	0.96	0.20	0.58	0.23	0.61	0.25	0.60	0.24
	Group B	0.96	0.08	0.62	0.13	0.60	0.22	0.56	0.18
	p-value	0.911		0.651		0.881		0.689	
Modified Gingival Index	Group A	1.18	0.32	0.62	0.22	0.55	0.23	0.61	0.35
	Group B	0.99	0.32	0.55	0.23	0.52	0.21	0.46	0.21
	p-value	0.098		0.903		0.694		0.174	

Independent t-test applied, p-value significant at p<0.05

**Table 2:** Intergroup and Intragroup changes in Plaque index and Gingival index at different time intervals.

Groups	Changes in Plaque Index						Changes in Modified Gingival Index					
	BL-3 month		BL-6 months		BL-12 months		BL-3 month		BL-6 months		BL-12 months	
	Mean	SD.	Mean	SD.	Mean	SD.	Mean	SD.	Mean	SD.	Mean	SD.
Group A	0.38	0.20	0.35	0.25	0.36	0.25	0.56	0.32	0.63	0.39	0.57	0.54
Group B	0.34	0.15	0.36	0.23	0.39	0.20	0.38	0.31	0.47	0.35	0.52	0.31
p-value	0.554		0.943		0.757		0.119		0.231		0.751	
Change in Group A (p-value)	0.0001		0.0001		0.0001		0.0001		0.0001		0.0001	
Change in Group B (p-value)	0.0001		0.0001		0.0001		0.0001		0.0001		0.0001	

Paired t-test applied, p-value significant at p<0.05

**Table 3:** Comparison of study groups with respect to Dummett Gupta Oral Pigmentation Index at different time intervals.

Groups	Baseline		3 months		6 months		12 months		BL-3 months		BL-6 months		BL-12 months	
	Mean	SD.	Mean	SD.	Mean	SD.	Mean	SD.	Mean	SD.	Mean	SD.	Mean	SD.
Group A	2.05	0.58	0.05	0.16	0.15	0.28	0.42	0.34	2.0	0.61	1.90	0.63	1.63	0.70
Group B	2.06	0.60	0.0	0.0	0.07	0.17	0.47	0.22	2.06	0.60	1.98	0.63	1.58	0.59
p-value	0.977		0.178		0.381		0.632		0.774		0.721		0.851	

Independent t-test applied, p-value significant at p<0.05

**Table 4:** Comparison of study groups with respect to VAS scores at different time intervals.

Groups	24 hours		7 days		24 hours- 7 days		Change in each group
	Mean	SD.	Mean	SD.	Mean	SD.	p-value
Group A	6.44	10.04	0.0	0.0	6.44	10.04	0.022
Group B	21.69	29.03	0.0	0.0	21.69	29.03	0.009
t-value	-1.986		-		3.500		-
p-value	0.056		-		0.001		-

Independent t-test applied, p-value significant at p<0.05
